# Neuromonitoring depth of anesthesia and its association with postoperative delirium

**DOI:** 10.1038/s41598-022-16466-y

**Published:** 2022-07-26

**Authors:** Berta Pérez-Otal, Cristian Aragón-Benedí, Ana Pascual-Bellosta, Sonia Ortega-Lucea, Javier Martínez-Ubieto, J. M. Ramírez-Rodríguez, Research Group in Anaesthesia, Resuscitation, and Perioperative Medicine of Institute for Health Research Aragón (ISS Aragón), Berta Pérez-Otal, Cristian Aragón-Benedí, Ana Pascual-Bellosta, Sonia Ortega-Lucea, Javier Martínez-Ubieto, Natividad Quesada-Gimeno, Luis Alfonso Muñoz-Rodríguez, Teresa Jiménez-Bernadó, Guillermo Pérez-Navarro, Alejandro Lucas-Luesma, Fernando Carbó-Espinosa, Mariana Hormigón-Ausejo, Jorge Luis Ojeda-Cabrera

**Affiliations:** 1 Department of Anesthesia, Resuscitation and Pain Therapy, Miguel Servet University Hospital, 50009 Zaragoza, Spain; 2 Department of Surgery, University of Zaragoza, 50001 Zaragoza, Spain; 3 Department of Health Sciences, University of Zaragoza, 50001 Zaragoza, Spain; 4 Department of Statistical Methods, University of Zaragoza, 50001 Zaragoza, Spain

**Keywords:** Neurological disorders, Risk factors, Neurological disorders, Comorbidities, Neurological manifestations

## Abstract

Delirium after surgery or Postoperative delirium (POD) is an underdiagnosed entity, despite its severity and high incidence. Patients with delirium require a longer hospital stay and present more postoperative complications, which also increases hospital costs. Given its importance and the lack of specific treatment, multifactorial preventive strategies are evidenced based. Our hypothesis is that using general anaesthesia and avoiding the maximum time in excessively deep anaesthetic planes through BIS neuromonitoring device will reduce the incidence of postoperative delirium in patients over the age of 65 and their hospitalization stay. Patients were randomly assigned to two groups: The visible BIS group and the hidden BIS neuromonitoring group. In the visible BIS group, the depth of anaesthesia was sustained between 40 and 60, while in the other group the depth of anaesthesia was guided by hemodynamic parameters and the Minimum Alveolar Concentration value. Patients were assessed three times a day by research staff fully trained during the 72 h after the surgery to determine the presence of POD, and there was follow-up at 30 days. Patients who developed delirium (n = 69) was significantly lower in the visible BIS group (n = 27; 39.1%) than in the hidden BIS group (n = 42, 60.9%; *p* = 0.043). There were no differences between the subtypes of delirium in the two groups. Patients in the hidden BIS group were kept for 26.6 ± 14.0 min in BIS values < 40 versus 11.6 ± 10.9 min (*p* < 0.001) for the patients in the visible BIS group. The hospital stay was lower in the visible BIS group 6.56 ± 6.14 days versus the 9.30 ± 7.11 days (*p* < 0.001) for the hidden BIS group, as well as mortality; hidden BIS 5.80% versus visible BIS 0% (*p* = 0.01). A BIS-guided depth of anaesthesia is associated with a lower incidence of delirium. Patients with intraoperative neuromonitoring stayed for a shorter time in excessively deep anaesthetic planes and presented a reduction in hospital stay and mortality.

## Introduction

The number of surgeries in older patients increases considerably over the years, leading to increased awareness of their susceptibility to postoperative neuropsychiatric complications, including postoperative delirium and cognitive impairment^1^.

While it is true that anaesthesia alone does not affect cognitive recovery in adults^2^, general anaesthesia is rarely found separately from a surgical act. There is a widespread belief that the effects of general anaesthesia accompanied by surgery are temporary, however recent studies suggest that surgical anaesthesia can have long-term effects on memory and perception. Researchers have found evidence of increased risk of cognitive and memory impairment in elderly patients following anaesthesia^3^.

Delirium is a complication with multifactorial aetiology and is frequent in advanced age patients. This entity has traditionally been regarded as a transient illness, however developing delirium in the early postoperative course is associated with long-term consequences, including increased mortality, prolonged hospitalization, discharge to an institution, and long-term cognitive decline and dementia^4^.

Delirium causes fluctuating changes in cognitive ability and alterations in perception, including hallucinations and behaviour alterations^5^. One of the main difficulties regarding delirium is the variability in its diagnosis, and they exist an important underdiagnosis, particularly for the hypoactive delirium group^1^.

The use of intraoperative neurological monitoring to guide the depth of anaesthesia facilitates the titration of anaesthetic drugs, reducing their doses by 11 to 27%^6^. Many studies have been conducted in recent years to find an association between anaesthetic depth through the analysis of electroencephalogram (EEG) waves and the incidence of POD^7–10^. It has been demonstrated that a high level of anaesthetic depth measured by the Bispectral Index (BIS) during a long period, as well as high doses of anaesthesia, can be factors predicting the development of postoperative complications^11^. The influence of the anaesthetic plane depth on the presence of some neurological complications, such as delirium, cognitive dysfunction and cerebrovascular accident during follow-up, has been put forward throughout the years^10,12,13^; however, the major ENGAGES trial^8^ and other recent meta-analyses^9,14^ have demonstrated some controversy in the relationship between anaesthetic depth and delirium, even going so far as to show that neuromonitoring of anaesthetic depth increases the incidence of delirium^8^.

Our study hypothesizes that the use of balanced general anaesthesia, avoiding the maximum time on excessively deep anaesthetic planes through brain monitoring guided by a device analysing EEG waves, will reduce the hospital stay and the incidence of delirium in patients over the age of 65.

## Methods

### Study design

An observational, prospective, randomized and single-centre study in > 65-year-old patients who have undergone a surgical intervention under general anaesthesia in the Miguel Servet University Hospital in Zaragoza (HUMS) from July 2019 to January 2020.

### Ethics

The study was first approved by the Regional Ethics Committee of Aragón (CEICA) as requested by regional guidelines. The study was conducted according to the Declaration of Helsinki, and written informed consent was obtained from all subjects.

### Participants

The patients recruited were those over the age of 65 who were to undergo surgery in the Departments of General Surgery, Vascular Surgery, Urology Surgery, Otorhinolaryngology and Maxillofacial Surgery, under general anaesthesia, with a hospital stay of at least 2 days.

### Inclusion/exclusion criteria

The patients selected had to meet the following criteria: (1) over the age of 65 who (2) would undergo major surgical interventions with a duration of two or more hours with (3) a postoperative hospital stay of at least 2 days (4) with a preoperative physical status between I and IV as classified by the American Society of Anaesthesiology (ASA) and (5) under balanced general anaesthesia maintained with halogenated gases.

The following patients were excluded from the study: those who (1) were not able to conduct the preoperative and postoperative interviews required by the study, (2) those who have communication difficulties, such as lack of understanding of the language, as well as high-grade hearing and visual disabilities, (3) patients with central nervous system diseases such as dementia or memory deterioration, with a score of 23 or lower in the mini-mental state examination (MMSE scale), (4) patients rejecting their inclusion in the study, and (5) patients not submitted to general anaesthesia because sedation in neuraxial or regional anaesthesia does not usually reach such deep anaesthetic planes (BIS < 40) as those analysed in this study.

### Description of outcomes

#### Postoperative delirium

Delirium is defined as an acute cognitive syndrome of lack of attention and disorganized thinking, which tends to fluctuate over time, and develops within hours or days^5^. When this disease occurs after surgery, we can call it postoperative delirium, however, surgery is not its only aetiology, because it has a multifactorial origin. This entity is a frequent complication, with an incidence ranging between 17 and 61%^11^ according to the type of procedure and postoperative destination; and also severe, because it increases morbimortality and the presence of other complications in patients after surgery^15^. In our study we analysed the potential association existing between developing POD and anaesthesia neuromonitoring using the BIS, as this is the monitor available in all operating rooms in our hospital.

From a clinical point of view, three subtypes of delirium can be differentiated: hyperactive delirium, hypoactive, and a third mixed subtype with both hyperactive and hypoactive symptoms. While hyperactive delirium is characterized by excessive activation symptoms, such as agitation and restlessness, hypoactive delirium presents a lack of response and immobility; therefore there is usually a delayed diagnosis in the latter, with clinical signs that go unnoticed. We collected patients with diagnoses for the three subtypes.

#### Bispectral index

BIS is a parameter developed through the bispectral analysis of EEG. This monitor analyses the pattern of brain wave frequencies (proportion of fast frequencies and proportion of slow frequencies) and transforms it into a dimensionless “anaesthetic depth” number: it estimates the degree of electric activity in the brain. It is a non-invasive method, as it is obtained by applying a specific sensor on the patient’s forehead, and its analysis is shown on a monitor alongside other parameters useful to confirm a correct assessment. The Bispectral Index is shown as a number from 0 to 100, from complete lack of EEG activity (0) to normal EEG activity: patient awake (100). This index is used clinically to assess the effect of anaesthetic drugs and titrate their dose. It is complemented by the monitor visualization of the EGG wave for the frontal area^16^.

Deep muscular relaxation is essential to avoid interferences in BIS monitoring because a muscular activity acknowledged by the monitor EMG will produce beta waves which could be falsely interpreted as brain activity and raise the monitor values.

Regarding the maintenance of anaesthesia, the use of propofol as the continuous infusion was excluded, as well as the use of ketamine, due to their production of beta waves which cause a falsely high value of BIS in deep anaesthetic planes; dexmedetomidine was also excluded due to its production of delta waves, leading to unreliable values of anaesthetic depth^17^.

BIS levels from 40 to 60 are considered an adequate anaesthetic depth for surgical intervention. Planes below 40 must be avoided due to the risk of potential neurological complications^10,12,13^, as well as over 60, due to the risk of intraoperative awakening, throughout the entire surgical intervention. In our study, we have considered those anaesthetic planes with a BIS value below 40 as excessively deep.

### Data collection

Before the surgical intervention, the attending anesthesiologist randomized the patient according to a computer-generated random group assignment, which is accessed through the hospital's intranet system. In this way, patients were electronically randomized and assigned to a study group: Visible BIS monitor group or Hidden BIS monitor group. Patients, surgeons and operating theatre nurses were blinded to the treatment identity.

In the operating room, a BIS Quatro sensor (Medtronic, Mansfield, MA) was applied to the forehead of each patient before anaesthetic induction, and they were subsequently connected to its monitor.

In the group with a visible monitor, the anesthesiologist adjusted the dose of anaesthetic and hypnotic drugs to achieve a BIS value between 40 and 60 from the commencement of anaesthesia to the end of surgery. A sound alarm was activated whenever the BIS number value was not within these values.

In those patients from the group with non-visible BIS monitor, the anaesthetic dose was titrated according to the MAC value and the hemodynamical parameters monitored. MAC is the acronym for Minimum Alveolar Concentration, which is the concentration at atmospheric pressure which suppresses motor response in 50% of individuals. In pure inhalation anaesthesia, it is required to reach MAC 1.2–1.3 to prevent movement in 95% of patients. MAC is reduced as age increases and with the addition of some drugs such as opioids, dexmedetomidine, muscle relaxants, magnesium sulphate or nitrous oxide. The optimum MAC adjustment for each patient was performed according to the clinical criteria of the anesthesiologist responsible and the patient’s status.

In this group, the monitor was placed in the same way as in the visible BIS group, but its numbers were not visible to the anesthesiologist. BIS values were recorded in 5-s intervals using a data acquisition program, the nursing staff could see them and record them, taking note of whether in that time interval there were any records in the graph above or below the desired values. Not all BIS monitors in the hospital allow BIS numbers to be checked after more than one hour, so to avoid data loss, the nursing staff saved them every 30 min. However, this 30-min recording was strictly careful and blinded to surgeons, other nursing staff and the anesthesiologist.

General anaesthesia was induced by using propofol (1–2 mg/kg) or etomidate (0.2–0.3 mg/kg) according to the patient’s circumstances, in combination with fentanyl (2 mcg/kg) or remifentanil (0.1 mcg/kg/min), followed by nondepolarizing muscle relaxants, rocuronium (0.6 mg/kg) or cisatracurium (0.15 mg/kg), to facilitate tracheal intubation. The type of muscle relaxant used was chosen by the anesthesiologist, and its administration was repeated according to the values in the TOF monitor, which was used to monitor all patients. Anaesthesia was maintained by volatile anaesthetics: desflurane or sevoflurane (MAC: 0.8–1%). Intraoperative analgesia was conducted through continuous remifentanil perfusion (0.1–0.25 mcg/kg/min) and fentanyl (1.5–2.5 mcg/kg) bolus injections according to the type of surgery.

Non-opioid drugs such as paracetamol 1 g, Enantyum 50 mg or metamizole 2 g, was routinely given 30 min before the end of surgery for postoperative pain management. Long-acting opioids (morphine 0.05–0.1 mg/kg) were chosen for major operations in combination with non-opioid medication.

Once the intervention was over, the patient was transferred to the Post Anesthesia Care Unit, Resuscitation Unit, or Intensive Care Unit, according to the postoperative care required, and their vital signs were recorded at arrival.

#### Delirium assessment

Patients were evaluated during the first 72 h after surgery, applying the DSM-5 criteria^5^ through the Confusion Assessment Method (CAM Scale) Annex 1. The first delirium assessment was in the Post Anesthesia Care Unit and then three times a day using the CAM or if the patients were in the ICU, the adapted CAM-ICU. All research staff were fully trained via the online training as per the guidelines for the CAM and CAM-ICU. POD assessments were performed only for Staff training was overseen by the principal autor and the Department of Psychiatry of the Hospital.

Delirium subtypes were classified according to the Richmond Agitation-Sedation Scale (RASS) Annex 2. The CAM-ICU scale was used in those patients who were unable to talk on these days and who had RASS values from − 2 to + 4. Assessors undertaking delirium and cognition assessments were blind to the allocation group.

Delirium is a fluctuating disorder, so to avoid overlooking the diagnosis of some patients when conducting interval assessments, CAM assessments were supplemented with an independent structured review of medical and nursing records for any potential delirium symptomatology. Mortality was recorded 30 days after the operation.

#### Sample size

Assuming a variable incidence of delirium between 44 and 81%^18^ in post-surgery patients over the age of 65 in our setting, with a significant level of 5% and a 90% of power, a sample size of 98 patients per group was calculated using the EPIDAT v. 4.1. software. Patient recruitment was performed until such sample was completed in a recruitment period from July 2019 to January 2020.

### Statistical analysis

To conduct a comparison between quantitative variables with normal distribution, we used the Student Fisher t-test and the analysis of variance (ANOVA) for the comparison between more than two means. For the comparison of quantitative variables with non-normal distribution, we applied the Mann–Whitney’s U test, as well as non-parametric ANOVA and Kruskal–Wallis as non-parametric tests.

To explain the value of a continuous variable based on the values taken by a series of explanatory variables, we applied a Linear Model. When the distribution of the values of variables was not normal, we applied the Generalized Linear Model (GLM). A multiple logistical regression analysis was used for the known risk factors: visible BIS, age, value in the BUPA scale Annex 3, duration of surgery, ASA physical status, delirium, and minutes of BIS below 40 with mortality.

Differences were considered significant when their *p* value was < 0.05. The different analyses were performed using commands from the basic “stats” package of Software “R” version 3.1.2.

## Results

**Figure 1:**
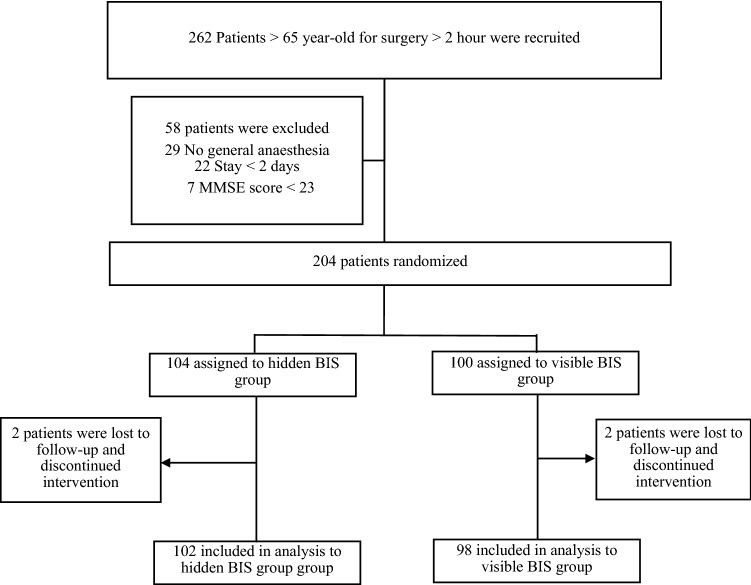
STROBE patient flow diagram. Screening, randomization, and the follow-up. BIS indicates bispectral index; *MMSE* mini-mental state examination.

During the study period, 262 patients were included, 58 of whom were excluded, as detailed in Fig. 1. STROBE patient flow diagram; 29 because they did not undergo surgery under general anaesthesia, 22 patients who did not stay hospitalized over 2 days, and 7 who obtained < 23 scores on the MMSE scale.

In total, 204 patients were included in the study, recruited consecutively. With a 2% drop-out rate, 200 patients in total completed the study (98 in the visible BIS group, 102 in the hidden BIS group), undergoing surgical interventions by one of the five surgical specialities included in our study, from July 2019 and until January 2020; they all met the inclusion criteria, and there was follow-up at 30 days for all of them.

All patients included in our study were over the age of 65, and the mean age of the patients collected was 75.38 years ± 6.52 years. Both groups were homogeneous, as detailed in Table 1.

**Table 1:** Homogeneity and comparison of demographic data and comorbidities between groups.

Homogeneity	Visible bis group N = 98	Hidden bis group N = 102	*P*
Age; years	74.99 ± 6.77	75.75 ± 6.3	0.388
Male	51.49% (52)	48.51% (49)	0.119
Female	46.46% (46)	53.53% (53)	
Weight; kg	71.9 ± 15.3	73.6 ± 13.2	0.265
ASA status: I and II	51.02% (50)	49.01% (50)	0.992
ASA status: III and IV	48.98% (48)	50.98% (52)	
Taking any antiaggregant or anticoagulant	42.85% (42)	46.07% (47)	0.058
Taking neuroleptic and antidepressive drugs	42.7% (35)	53.4% (63)	0.136
Number of drugs prescribed	4.49 ± 3.03	5.00 ± 2.84	0.149
**Preexisting medical conditions**			
Cardiovascular	67.3% (66)	76.5% (78)	0.151
Respiratory	31.6% (31)	36.3% (37)	0.489
Endocrinological-renal	77.6% (76)	76.5% (78)	0.856
Neurological	35.7% (35)	40.2% (41)	0.514

Patient characteristics at entry of the trial.

Values are number (%) or mean ± SD.

ASA indicates patients' scores on the ASA anaesthesia risk assessment scale.

## Anesthesia neuromonitoring

Delirium was detected in 69 patients (34.5%). Of these, 39.1% (n = 27) were from the visible BIS group, while 60.9% (n = 42) belonged to the hidden BIS group (*p* = 0.043). Patients from non-visible BIS group had a higher incidence of hypoactive delirium (30.4%) versus visible BIS (16.3%), but these were not statistically significant (*p* = 0.053). There were also no statistically significant differences for the hyperactive subtype (11.8% vs. 11.2%; *p* > 0.05).

**Figure 2:**
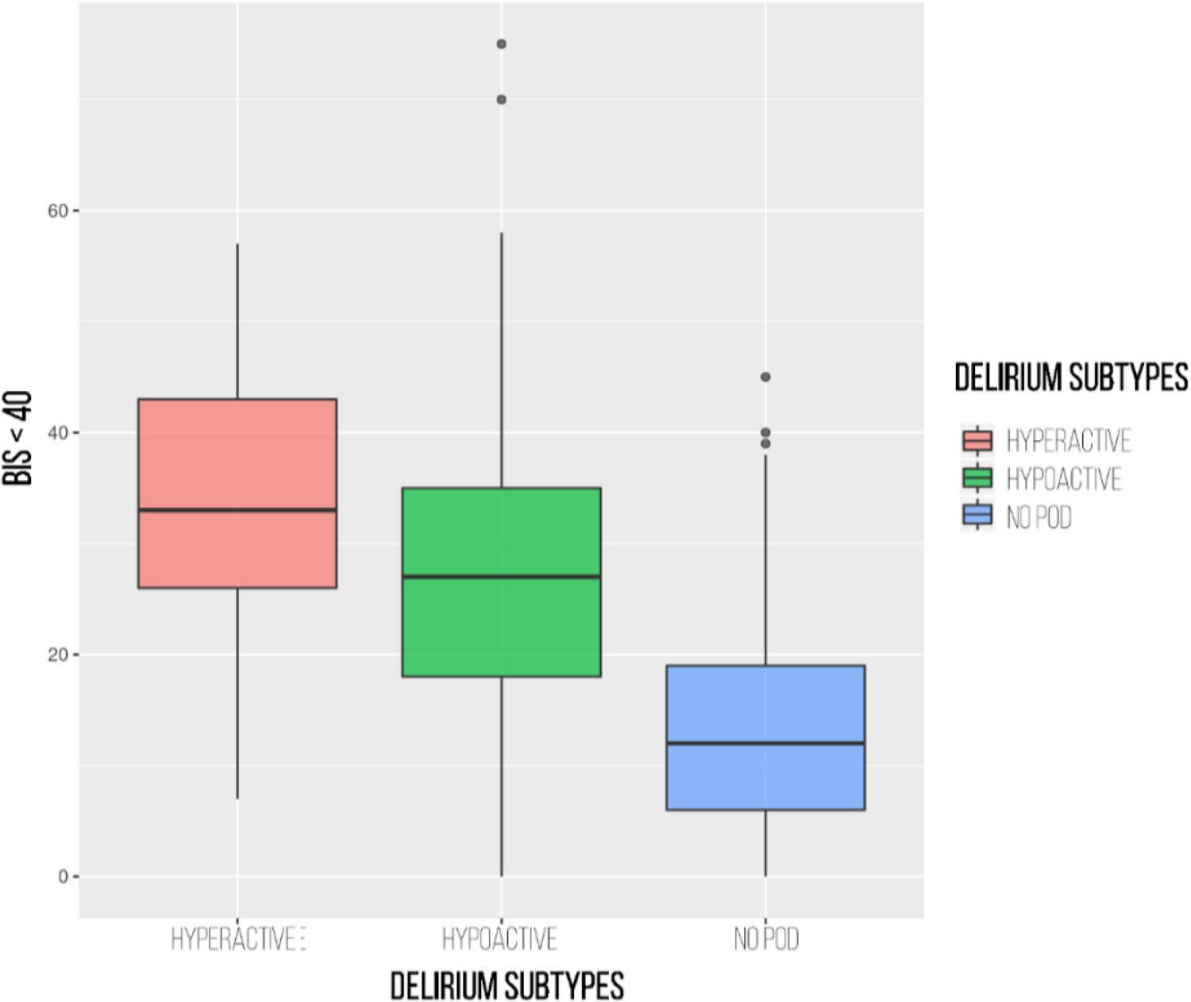
Incidence of postoperative delirium and its relationship with the minutes of anaesthesia depth. BIS < 40 indicates the time in minutes that the BIS value has been below 40.

Patients with delirium were kept with BIS values below 40 for a mean of 30.46 ± 15.88 min versus 13.37 ± 9.71 min for patients who did not develop this neurological complication (*p* < 0.001). Those patients who presented hyperactive POD stayed for a mean of 33.65 min ± 13.67 min with BIS < 40 versus 28.9 ± 16.61 min for patients who presented hypoactive POD (*p* < 0.001) (Fig. 2).

Besides, those patients from the non-visible BIS group stayed a longer time with a BIS below 40: a mean of 26.6 ± 14.0 min versus 11.6 ± 10.9 min (*p* < 0.001) for patients in the visible BIS group.

In terms of hospital stay, patients from the non-visible BIS group presented a stay in the hospitalization ward of 9.30 ± 7.11 days versus 6.56 ± 6.14 days for patients from the visible BIS group (*p* < 0.001).

Mortality at 30 days was 5.80% (n = 4) in the non-visible BIS group; all patients had presented delirium within the first 72 h; on the other hand, no patients died in the visible BIS group. Two of the patients who died had presented hyperactive delirium, while the other two presented hypoactive delirium. Anaesthetic depth was not directly associated with death in any of these cases (massive haemorrhage, multiorgan failure, and two cases of malignant tumour relapse).

## Postoperative delirium

34.5% (n = 69) of patients presented delirium; 32.9% (n = 23) of them developed hyperactive or mixed delirium, while the remaining 67.1% (n = 46) suffered hypoactive delirium. Patients with delirium had a significantly higher mean age than patients without (77.32 ± 6.50 vs. 74.36 ± 6.33; *p* = 0.002). The incidence of delirium increased with age; the incidence of delirium was 40% in patients aged 75 years or older (n = 42) and 63.16% (n = 12) of patients over 85 years. Patients who developed delirium presented also statistically significant differences regarding ASA classification status, presence of previous neurological background, and drugs are taken (Table 2).

**Table 2:** Preoperative risk factors for developing postoperative delirium at 72 h.

	POD at 72 h	No POD at 72 h	*P*
No. patients (n)	69	131	
Age; years	77.32 ± 6.5	74.36 ± 6.3	0.002
Male	52.2% (36)	49.6% (65)	0.731
Female	47.8% (33)	50.4% (66)	
Weight; kg	72.6 ± 13.6	72.8 ± 14.7	0.944
ASA I and II	36.23% (25)	57.26% (75)	0.009
ASA III and IV	63.77% (44)	42.74% (56)	
Taking any antiaggregant and/or anticoagulant	66.7% (46)	38.93% (51)	< 0.001
Taking neuroleptic and/or antidepressant drugs	55.1% (38)	33.6% (44)	0.003
Polypharmacy (number of drugs prescribed)	6.10 ± 2.93	4.04 ± 2.70	< 0.001
**Preexisting medical conditions**			
Cardiovascular	79.7% (55)	67.9% (89)	0.078
Respiratory	39.1% (27)	31.3% (41)	0.266
Endocrinological-renal	81.2% (56)	74.8% (98)	0.310
Neurological	53.6% (37)	29.8% (39)	< 0.001

Values are number (%) or mean ± SD.

POD indicates postoperative delirium.

The mean duration of surgical interventions was 178 ± 83.3 min. Patients with delirium stayed longer under general anaesthesia 224.9 ± 105.2 min compared to patients without delirium 153 ± 55.2 min (*p* < 0.001). Delirium occurred more frequently in complex surgical interventions and decreases order according to the complexity of the surgery. According to the BUPA scale (Annex 3), delirium occurred in 26 (57.78%) patients undergoing Complex Plus surgery, 32 (39.02%) patients undergoing Major Plus surgery, 10 (16.39%) patients undergoing Major surgery and only one (8.33%) patient undergoing surgery with Intermediate complexity.

82 patients (41%) required admission to the Intensive Care Unit (ICU). The incidence of delirium was statistically significant higher (58.0%) in them than in the Post Anaesthesia Recovery Unit patients (42.0%; *p* < 0.001). The ICU stay of patients with delirium was significantly longer (2.45 ± 5.63 vs. 0.37 ± 0.65; *p* < 0.001) as well as the total hospital stay (14.99 ± 8.46 vs. 5.98 ± 4.71; *p* < 0.001).

## Discussion

This is the first observational, prospective, randomized, double-blind and single-centre study in patients over the age of 65 undergoing surgery by different surgical specialities under general anaesthesia, in the Miguel Servet University Hospital, to find a potential association between the incidence of postoperative delirium and the existence of excessively deep anaesthetic planes during the intraoperative period.

The main studies about the incidence of delirium and its association with anaesthetic depth have been conducted by Radtke^19^ in Germany, Chan^10^ in Hong Kong, who conducted the Cognitive Dysfunction after Anesthesia (CODA) study, Wildes^8^ in the U.S.A., who led the major study Electroencephalography Guidance of Anesthesia to Alleviate Geriatric Syndromes (ENGAGES) and the recent study of Evered^7^ in 2021. Three important meta-analyses by Miao^9^, Jannsen^11^ and Shan^14^ should also be noted.

While Evered^7^, Chan^10^ and Radtke^19^ and showed a statistically significant reduction in the incidence of delirium among patients with BIS-guided anaesthetic depth, the meta-analyses by Miao and Jannsen found no statistically significant association and the study by Wildes et al.^8^, conducted in 1213 patients, showed a 3% higher incidence of delirium in the neuromonitoring group.

Our study demonstrated that limited exposure to anaesthetic drugs avoiding the excessively deep planes identified by BIS < 40 led to a significant reduction in postoperative delirium (27.55% vs. 41.17%; p=0.043). This should facilitate rehabilitation in short term and functional recovery in long term as Evered’s article has shown^7^.

The incidence of delirium in our study was 34.5%; within this proportion, 67.1% presented hypoactive delirium and 32.9% presented mixed or hyperactive delirium. This incidence is slightly higher than the one found in the studies by Radtke^19^ (16.7–21.4%) and the CODA study^10^ (15.6% vs. 24.1%), two of the studies with more similarities to ours.

There was no differentiation in delirium diagnosis in any of the studies previously mentioned^8,10,19^ and many of them only collected the cases with hyperactive or mixed subtypes, which could explain their lower incidence outcomes than those found in our study, where the hypoactive subtype presented the highest incidence.

Even though the hyperactive type is easier to diagnose due to its striking symptomatology, this only represents 20% of the total, while hypoactive delirium represents over 50%, but is the most underdiagnosed subtype due to its characteristics^11^. An early diagnosis is essential for this subtype because it presents a worse prognosis, probably due to a delay in treatment and its high mortality.

On the other hand, our study population consisted mainly of patients belonging to the ASA II and ASA III groups (46% and 45% respectively), while 5% of patients were in the ASA IV group. Of those patients presenting delirium, 63.77% were from the ASA III and IV groups, demonstrating that there is a statistical association between delirium and the ASA score.

Unlike in our study, patients in these two previous studies^10,19^ presented lower ASA scores, younger age, and underwent shorter surgical procedures; this could justify the difference in the incidence of POD between our study and the CODA study^10^, given that our patients had a higher anaesthetic risk, were older, and presented higher surgical complexity, and these parameters have been statistically associated with the presence of POD.

41% patients required postoperative care in a Critical Care Unit. This high proportion can be caused by the characteristics of patients included in our study, with a higher mean age (entailing a higher number of comorbidities, and therefore a higher score on the ASA scale) and selecting surgical procedures with a minimum duration over two hours and with higher complexity versus other similar studies^8,10,19^.

The incidence of POD was statistically higher (58%) in those patients admitted to the Resuscitation Unit or ICU than in those transferred to the Post-Anesthesia Recovery Unit (PARU) (42%). This outcome is consistent with literature, because the risk of developing POD in the ICU is 7 times higher than in the hospitalization ward^11^. The use of invasive measures, such as intravascular catheters, is more frequent in these units than in the hospitalization wards. These catheters, particularly central lines and arterial catheters, have been associated with an increase in the incidence of POD.

Our main finding was that neuromonitoring the depth of anaesthesia through BIS reduced the incidence of delirium in advanced-age patients. Our study demonstrated that the incidence of postoperative delirium in patients over the age of 65 undergoing surgery was significantly lower in those cases where excessively deep anaesthetic planes (BIS < 40) were avoided.

In terms of hospital stay, patients who developed delirium had, in absolute terms, a statistically longer hospital stay than those who did not present it. These results are consistent with others published in the literature^7,20–22^.

As literature reviewed^10^, BIS-guided anaesthetic depth is also associated with hospital stay; in our study, patients in the non-visible BIS group stayed more days in hospital than those in the visible BIS group.

Our explanation for this increase in hospital stay might be that patients with delirium are less likely to participate in postoperative pulmonary rehabilitation and early mobilization, which generates a high risk of developing postoperative complications such as pneumonia, deep vein thrombosis and cerebrovascular accidents with neurological impairment, as confirmed by the reviewed literature^23,24^.

There was a 5.80% mortality in our study; there was no mortality at 30 days in the visible BIS group, while 4 patients from the non-visible BIS group died, in all cases after experiencing delirium. Death was not directly associated with depth of anaesthesia in any of these cases.

The B-Aware study showed higher mortality in patients submitted to deep anaesthesia, defined as BIS < 40, up to 4–1 years after the surgical procedure^13^. The deep anaesthesia episodes were also associated with a higher risk of myocardial infarction and cerebrovascular accident during follow-up. These outcomes coincide with those in our study, which showed that patients presenting postoperative complications stayed on BIS < 40 for a statistically higher cumulative period than those who did not present them (14.6 ± 11.7 min vs. 26.6 ± 15.9 min).

In conclusion, an adequate depth of anaesthesia through intraoperative neuromonitoring, as well as a carefully titrated anaesthesia dosing, can prevent non-intentional deep anaesthetic planes, and could be useful for improving the postoperative cognitive performance in the elderly, their postoperative recovery, mortality, and to achieve a shorter hospital stay^7,25^. Some renowned guidelines^26,27^ and recent meta-analyses^11^ recommend monitoring with intraoperative EEG to avoid an excessive administration of anaesthetic drugs to patients at high risk of postoperative delirium within a multicomponent intervention.

## Limitations

Our study presents some limitations. In the first place, it was a single-centre study conducted at a Tertiary Centre of Reference, and our findings can be associated with the characteristics of our elective surgery population and, therefore, not necessarily applicable to other centres.

There are also specific limitations with BIS monitoring during clinical anaesthesia. The BIS monitor does not provide a number that can be interpreted in isolation from its clinical context. Different environmental and physiological factors can have an impact on BIS performance. Electric electrocardiographic and electromyographic devices, and those for electrocautery, with 50 Hz network interference, introduce high-frequency signals and are the main source of errors^28^.

In third place, delirium can be difficult to diagnose, as there are no objective biomarker tests. Scores in our tests for POD diagnosis cannot be directly compared with other studies because different tests were used. We chose sensitive tests which have been validated for our local population, such as the CAM scale and clinical assessment.

The highest sensitivity for the diagnosis of postoperative diagnosis is achieved through the application of the Nu-DESC (Nursing Delirium Screening Scale) and CAM (Confusion Assessment Method) scales whose sensitivity decreases when not applied by properly trained staff. In our study, all research staff were fully trained as per the guidelines for the CAM. Besides, the incidence of delirium found in our study was similar to that found in other studies reviewed, and therefore we believe that our methods for diagnosis have been adequately applied.

## Conclusions

Based on the outcomes of our study, we can draw the conclusion that those patients over 65 years of age with a depth of anaesthesia not guided by brain neuromonitoring present a higher incidence of delirium and a longer hospital stay in our setting. On the other hand, those patients with monitored depth of anaesthesia will remain for a shorter time in deep anaesthetic planes (BIS < 40) and present a shorter stay at the hospital and lower mortality.

## Supplementary Information

Supplementary Information.

## Data Availability

The datasets used and analyzed during the current study are available from the corresponding author on reasonable request.
