# Supplementary material for: Neuromonitoring depth of anesthesia and its association with postoperative delirium
Source: Sci Rep. 2022 Jul 26;12:12703. doi: 10.1038/s41598-022-16466-y (PMC9325758; doi:10.1038/s41598-022-16466-y)
Supplement: Supplementary file 1 — Supplementary Information. [file 41598_2022_16466_MOESM1_ESM.docx]

**SUPPLEMENTARY MATERIAL**

**ANNEX 1**: DSM-V criteria for the diagnosis of delirium.

| **A. Alteration of awareness (e.g., reduction in attention to the environment) with a reduction in the ability to focus, sustain or shift attention.** | **A + B**  **and/or**  **C**  **and/or** |
| --- | --- |
| **B. A change in cognitive functions (such as memory deficit, disorientation, language alteration), or presence of a perceptive alteration that cannot be explained by the existence of previous dementia under development.** |  |
| **C. The alteration develops within a short period (typically in hours or days) and tends to fluctuate during the day.** |  |
| **D. There is evidence from the history, physical examination, and lab tests, that the alteration is a direct physiological effect of a medical condition, due to the use of medications or intoxication by some substance, abstinence syndrome, or multiple or non-specified etiologies.** | **D**  **=**  **DELIRIUM** |

**ANNEX 2**: Richmond Agitation Sedation Scale (RASS) for diagnosis of postoperative delirium subtypes..

| **Score** | **TERM** | **DESCRIPTION** | **EXAMINATION** |
| --- | --- | --- | --- |
| **+4** | **Combative** | Combative, violent, with immediate danger to staff. | Watch the patient. |
| **+3** | **Very agitated** | Aggressive; tries to remove tubes and catheters. |  |
| **+2** | **Agitated** | Frequent non-purposeful movement, “fights” the ventilator. |  |
| **+1** | **Restless** | Anxious, but without aggressive or vigorous movements. |  |
| **0** | **Alert and calm** |  |  |
| **-1** | **Drowsy** | Not fully alert but has sustained awakening (> 10 seconds) when called. | Call the patient by their name and ask them to open their eyes and look at us. |
| **-2** | **Mild sedation** | Briefly awakens (<10 s) with eye contact when called. |  |
| **-3** | **Moderate sedation** | Movement or eye opening when called, but no eye contact. |  |
| **-4** | **Deep sedation** | No response when called, but movement or eye opening to physical stimulation. | Stimulate the patient by shaking their shoulder or rubbing their sternum. |
| **-5** | **No response** | No response to voice or physical stimulation. |  |
| **If RASS value is -4 or -5, stop and assess the patient again afterwards.**  **If RASS value is over -4, then proceed to delirium assessment, if indicated.** | | | |

# ANNEX 3: BUPA Scale. Surgery Scale according to the classification by the British United Provident Association (BUPA):

- 1. Minor: 1
  2. Intermediate: 2
  3. Major: 3
  4. Major plus: 4
  5. Complex plus: 5
